# 4′-[5-(4-Fluoro­phen­yl)pyridin-3-yl]-1′-methyl­dispiro­[indan-2,2′-pyrrolidine-3′,2′′-indan]-1,3,1′′-trione

**DOI:** 10.1107/S1600536811032946

**Published:** 2011-08-27

**Authors:** Ang Chee Wei, Mohamed Ashraf Ali, Rusli Ismail, Ching Kheng Quah, Hoong-Kun Fun

**Affiliations:** a Institute for Research in Molecular Medicine, Universiti Sains Malaysia, 11800 USM, Penang, Malaysia; b X-ray Crystallography Unit, School of Physics, Universiti Sains Malaysia, 11800 USM, Penang, Malaysia

## Abstract

In the title compound, C_32_H_23_FN_2_O_3_, the pyrrolidine ring adopts an envelope conformation. The monoketo- and diketo-substituted five-membered rings are in envelope and half-chair conformations, respectively. The mol­ecular structure is stabilized by an intra­molecular C—H⋯O hydrogen bond, which generates an *S*(6) ring motif. In the crystal, mol­ecules are linked *via* inter­molecular C—H⋯N and C—H⋯O hydrogen bonds into a three-dimensional network. The crystal structure is further consolidated by C—H⋯π inter­actions.

## Related literature

For general background to and the biological activity of the title compound, see: Chande *et al.* (2005 ▶); Prasanna *et al.* (2010 ▶); Karthikeyan *et al.* (2010 ▶); Dye (2002 ▶); Duncan & Barry (2004 ▶). For related structures, see: Kumar *et al.* (2010 ▶); Wei *et al.* (2011 ▶). For reference bond-length data, see: Allen *et al.* (1987 ▶). For hydrogen-bond motifs, see: Bernstein *et al.* (1995 ▶). For the stability of the temperature controller used in the data collection, see: Cosier & Glazer (1986 ▶). For ring conformations, see: Cremer & Pople (1975 ▶).
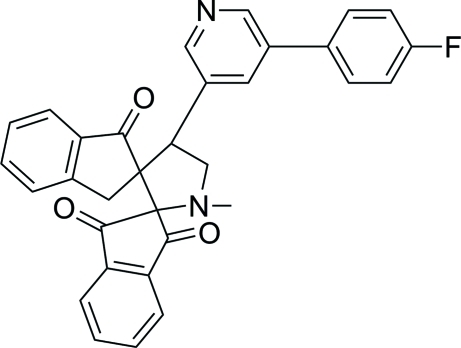


## Experimental

### 

#### Crystal data

C_32_H_23_FN_2_O_3_
*M* *_r_* = 502.52
Monoclinic, 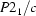

*a* = 14.8997 (2) Å
*b* = 7.7993 (1) Å
*c* = 23.0188 (3) Å
β = 112.638 (1)°
*V* = 2468.86 (6) Å^3^
*Z* = 4
Mo *K*α radiation
μ = 0.09 mm^−1^
*T* = 100 K
0.36 × 0.17 × 0.05 mm

#### Data collection

Bruker SMART APEXII CCD area-detector diffractometer
Absorption correction: multi-scan (*SADABS*; Bruker, 2009 ▶) *T*_min_ = 0.968, *T*_max_ = 0.996
27325 measured reflections
7184 independent reflections
4664 reflections with *I* > 2σ(*I*)
*R*_int_ = 0.057

#### Refinement

*R*[*F* ^2^ > 2σ(*F* ^2^)] = 0.053
*wR*(*F* ^2^) = 0.135
*S* = 0.99
7184 reflections
344 parameters
H-atom parameters constrained
Δρ_max_ = 0.29 e Å^−3^
Δρ_min_ = −0.27 e Å^−3^

### 

Data collection: *APEX2* (Bruker, 2009 ▶); cell refinement: *SAINT* (Bruker, 2009 ▶); data reduction: *SAINT*; program(s) used to solve structure: *SHELXTL* (Sheldrick, 2008 ▶); program(s) used to refine structure: *SHELXTL*; molecular graphics: *SHELXTL*; software used to prepare material for publication: *SHELXTL* and *PLATON* (Spek, 2009 ▶).

## Supplementary Material

Crystal structure: contains datablock(s) global, I. DOI: 10.1107/S1600536811032946/wn2449sup1.cif

Structure factors: contains datablock(s) I. DOI: 10.1107/S1600536811032946/wn2449Isup2.hkl

Additional supplementary materials:  crystallographic information; 3D view; checkCIF report

## Figures and Tables

**Table 1:** Hydrogen-bond geometry (Å, °) *Cg*1 is the centroid of the N2/C22–C26 pyridyl ring.

*D*—H⋯*A*	*D*—H	H⋯*A*	*D*⋯*A*	*D*—H⋯*A*
C6—H6*A*⋯N2^i^	0.95	2.56	3.476 (2)	163
C13—H13*A*⋯N1^ii^	0.95	2.45	3.387 (2)	168
C15—H15*A*⋯O2^iii^	0.95	2.44	3.381 (3)	169
C18—H18*A*⋯O2	0.99	2.40	3.077 (2)	125
C20—H20*A*⋯O3^iv^	0.99	2.55	3.318 (2)	135
C4—H4*A*⋯*Cg*1^v^	0.95	2.65	3.552 (2)	159

Symmetry codes: (i) 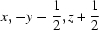; (ii) 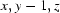; (iii) 
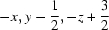
; (iv) 
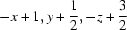
; (v) 
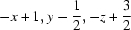
.

**Fig. 1.**
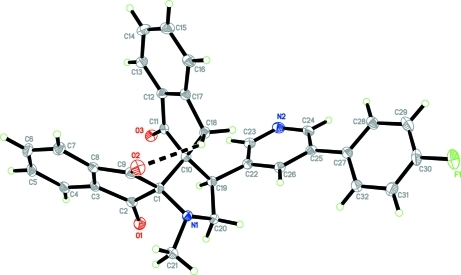
The molecular structure of the title compound showing 50% probability displacement ellipsoids for non-H atoms. The intramolecular hydrogen bond is shown as a dashed line.

**Fig. 2.**
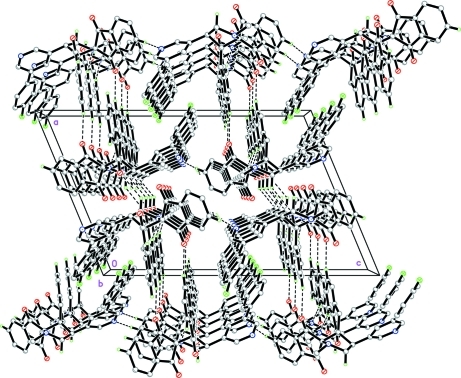
The crystal structure of the title compound, viewed along the b axis. H atoms not involved in hydrogen bonds (dashed lines) have been omitted for clarity.

## supplementary crystallographic information

### Comment

Spiro compounds have drawn considerable attention due to their antiseptic, analgesic and broad-spectrum antimicrobial activities (Chande *et al.*, 2005). Many of these compounds are found to have comparable or even better antimycobacterial activities than some of the first-line TB drugs currently available (Prasanna *et al.*, 2010; Karthikeyan *et al.*, 2010). Tuberculosis (TB) is a chronic illness caused by Mycobacterium tuberculosis and kills approximately 2 million people each year (Dye, 2002). Thus there is an urgent need to develop potent new antitubercular agents with new mechanisms of action and low toxicity profiles which are effective against drug-susceptible and drug-resistant strains of M. tuberculosis (Duncan & Barry, 2004).

The molecular structure is shown in Fig. 1. Bond lengths (Allen *et al.*, 1987) and angles are within normal ranges and are comparable to those in related crystal structures (Kumar *et al.*, 2010; Wei *et al.*, 2011).

The pyrrolidine ring (N1/C1/C10/C19/C20) adopts an envelope conformation, puckering parameters (Cremer & Pople, 1975) Q = 0.4855 (18) Å and φ = 41.8 (2)°, with atom C1 at the flap. The five-membered carbocyclic ring, C1-C3/C8/C9 is twisted on C9—C1, with puckering parameters Q = 0.1846 (18) Å and φ = 170.4 (6)°, thereby adopting a half-chair conformation. The five-membered carbocyclic ring, C10-C12/C17/C18 adopts an envelope conformation, with puckering parameters Q = 0.1277 (18) Å and φ = 359.4 (8)°, and with atom C10 at the flap.

If the six-membered rings N2/C22–C26, C3–C8, C12–C17 and C27–32 are denoted by R4, R5, R6, R7 then the dihedral angles for R4^R5, R5^R6, R4^R6 and R4^R7 are 69.10 (8), 39.95 (8), 78.82 (9) and 33.46 (9)°, respectively.

The molecular structure is stabilized by an intramolecular C18–H18A···O2 hydrogen bond (Table 1), which generates an *S*(6) ring motif (Fig. 1, Bernstein *et al.*, 1995). In the crystal structure, Fig. 2, molecules are linked *via* intermolecular C6—H6A···N2, C13—H13A···N1, C15—H15A···O2 and C20—H20A···O3 hydrogen bonds (Table 1) into a three-dimensional network. The crystal structure is further consolidated by C4—H4A···Cg1 (Table 1) interactions, where Cg1 is the centroid of the N2/C22-C26 pyridyl ring.

### Experimental

A mixture of (*E*)-2-(5-(4-flurophenyl) pyridin-3-yl) methylene)-2, 3-dihydro-*1H*-indene-1-one (0.001 mmol), ninhydrin (0.001 mmol) and sarcosine (0.002 mmol) (1:1:2) was dissolved in methanol (10 ml) and refluxed for 4 h. After completion of the reaction, as evident from TLC, the mixture was poured into water (50 ml). The precipitated solid was filtered, washed with water and recrystallised from a pet. ether - ethyl acetate mixture (1:1) to yield the title compound as yellow crystals.

### Refinement

All H atoms were positioned geometrically and refined using a riding model with C—H = 0.95-1.00 Å and *U*_iso_(H) = 1.2 or 1.5 *U*_eq_(C). A rotating-group model was applied for the methyl group.

### Crystal data

**Table:**

C_32_H_23_FN_2_O_3_	*F*(000) = 1048
*M**_r_* = 502.52	*D*_x_ = 1.352 Mg m^−^^3^
Monoclinic, *P*2_1_/*c*	Mo *K*α radiation, λ = 0.71073 Å
Hall symbol: -P 2ybc	Cell parameters from 5035 reflections
*a* = 14.8997 (2) Å	θ = 2.8–28.1°
*b* = 7.7993 (1) Å	µ = 0.09 mm^−^^1^
*c* = 23.0188 (3) Å	*T* = 100 K
β = 112.638 (1)°	Plate, yellow
*V* = 2468.86 (6) Å^3^	0.36 × 0.17 × 0.05 mm
*Z* = 4	

### Data collection

**Table:**

Bruker SMART APEXII CCD area-detector diffractometer	7184 independent reflections
Radiation source: fine-focus sealed tube	4664 reflections with *I* > 2σ(*I*)
graphite	*R*_int_ = 0.057
φ and ω scans	θ_max_ = 30.1°, θ_min_ = 1.9°
Absorption correction: multi-scan (*SADABS*; Bruker, 2009)	*h* = −16→20
*T*_min_ = 0.968, *T*_max_ = 0.996	*k* = −10→10
27325 measured reflections	*l* = −32→32

### Refinement

**Table:**

Refinement on *F*^2^	Primary atom site location: structure-invariant direct methods
Least-squares matrix: full	Secondary atom site location: difference Fourier map
*R*[*F*^2^ > 2σ(*F*^2^)] = 0.053	Hydrogen site location: inferred from neighbouring sites
*wR*(*F*^2^) = 0.135	H-atom parameters constrained
*S* = 0.99	*w* = 1/[σ^2^(*F*_o_^2^) + (0.0641*P*)^2^] where *P* = (*F*_o_^2^ + 2*F*_c_^2^)/3
7184 reflections	(Δ/σ)_max_ = 0.001
344 parameters	Δρ_max_ = 0.29 e Å^−^^3^
0 restraints	Δρ_min_ = −0.27 e Å^−^^3^

### Special details

**Table:**

Experimental. The crystal was placed in the cold stream of an Oxford Cryosystems Cobra open-flow nitrogen cryostat (Cosier & Glazer, 1986) operating at 100.0 (1) K.
Geometry. All esds (except the esd in the dihedral angle between two l.s. planes) are estimated using the full covariance matrix. The cell esds are taken into account individually in the estimation of esds in distances, angles and torsion angles; correlations between esds in cell parameters are only used when they are defined by crystal symmetry. An approximate (isotropic) treatment of cell esds is used for estimating esds involving l.s. planes.
Refinement. Refinement of F^2^ against ALL reflections. The weighted R-factor wR and goodness of fit S are based on F^2^, conventional R-factors R are based on F, with F set to zero for negative F^2^. The threshold expression of F^2^ > 2sigma(F^2^) is used only for calculating R-factors(gt) etc. and is not relevant to the choice of reflections for refinement. R-factors based on F^2^ are statistically about twice as large as those based on F, and R- factors based on ALL data will be even larger.

### Fractional atomic coordinates and isotropic or equivalent isotropic displacement parameters (Å^2^)

**Table:**

	*x*	*y*	*z*	*U*_iso_*/*U*_eq_	
F1	−0.02526 (8)	0.88163 (14)	0.42880 (5)	0.0352 (3)	
O1	0.53116 (8)	0.12559 (15)	0.85281 (6)	0.0203 (3)	
O2	0.22158 (9)	0.20148 (15)	0.85535 (6)	0.0212 (3)	
O3	0.38943 (8)	−0.17977 (14)	0.76020 (6)	0.0187 (3)	
N1	0.35687 (10)	0.35355 (16)	0.80293 (6)	0.0142 (3)	
N2	0.30185 (11)	0.07049 (18)	0.54724 (7)	0.0200 (3)	
C1	0.35388 (12)	0.17247 (19)	0.81705 (7)	0.0133 (3)	
C2	0.45362 (12)	0.08860 (19)	0.85599 (8)	0.0146 (3)	
C3	0.43827 (12)	−0.03372 (19)	0.90018 (7)	0.0144 (3)	
C4	0.50353 (13)	−0.1516 (2)	0.93975 (8)	0.0180 (4)	
H4A	0.5677	−0.1626	0.9409	0.022*	
C5	0.47110 (14)	−0.2526 (2)	0.97738 (8)	0.0206 (4)	
H5A	0.5142	−0.3338	1.0050	0.025*	
C6	0.37646 (14)	−0.2379 (2)	0.97576 (8)	0.0232 (4)	
H6A	0.3560	−0.3103	1.0015	0.028*	
C7	0.31210 (13)	−0.1179 (2)	0.93668 (8)	0.0202 (4)	
H7A	0.2481	−0.1062	0.9357	0.024*	
C8	0.34458 (12)	−0.0158 (2)	0.89913 (7)	0.0153 (3)	
C9	0.29442 (12)	0.1276 (2)	0.85693 (8)	0.0158 (3)	
C10	0.30739 (11)	0.09841 (19)	0.74827 (8)	0.0130 (3)	
C11	0.31431 (12)	−0.09919 (19)	0.74843 (7)	0.0140 (3)	
C12	0.21613 (12)	−0.1679 (2)	0.73393 (8)	0.0155 (3)	
C13	0.18807 (12)	−0.3386 (2)	0.73650 (8)	0.0178 (4)	
H13A	0.2347	−0.4285	0.7487	0.021*	
C14	0.09024 (13)	−0.3714 (2)	0.72068 (9)	0.0224 (4)	
H14A	0.0692	−0.4855	0.7225	0.027*	
C15	0.02184 (13)	−0.2386 (2)	0.70207 (9)	0.0225 (4)	
H15A	−0.0451	−0.2643	0.6909	0.027*	
C16	0.05016 (12)	−0.0699 (2)	0.69970 (8)	0.0191 (4)	
H16A	0.0033	0.0196	0.6867	0.023*	
C17	0.14895 (12)	−0.0346 (2)	0.71677 (7)	0.0150 (3)	
C18	0.19694 (11)	0.1362 (2)	0.71821 (8)	0.0155 (3)	
H18A	0.1781	0.2198	0.7439	0.019*	
H18B	0.1785	0.1826	0.6751	0.019*	
C19	0.37211 (12)	0.19184 (19)	0.71845 (8)	0.0137 (3)	
H19A	0.4335	0.1243	0.7297	0.016*	
C20	0.39874 (12)	0.3643 (2)	0.75442 (8)	0.0156 (3)	
H20A	0.4702	0.3790	0.7741	0.019*	
H20B	0.3704	0.4618	0.7257	0.019*	
C21	0.40153 (13)	0.4657 (2)	0.85715 (8)	0.0187 (4)	
H21A	0.3638	0.4615	0.8838	0.028*	
H21B	0.4026	0.5836	0.8427	0.028*	
H21C	0.4682	0.4275	0.8814	0.028*	
C22	0.32635 (12)	0.2035 (2)	0.64733 (8)	0.0150 (3)	
C23	0.33944 (12)	0.0714 (2)	0.61049 (8)	0.0171 (3)	
H23A	0.3775	−0.0243	0.6315	0.021*	
C24	0.24663 (12)	0.2045 (2)	0.51798 (8)	0.0185 (4)	
H24A	0.2200	0.2059	0.4733	0.022*	
C25	0.22608 (12)	0.3425 (2)	0.54969 (8)	0.0157 (3)	
C26	0.26802 (12)	0.3398 (2)	0.61536 (8)	0.0157 (3)	
H26A	0.2566	0.4323	0.6385	0.019*	
C27	0.16180 (12)	0.4862 (2)	0.51584 (8)	0.0170 (3)	
C28	0.08191 (13)	0.4584 (2)	0.45942 (8)	0.0207 (4)	
H28A	0.0704	0.3471	0.4412	0.025*	
C29	0.01936 (13)	0.5917 (2)	0.42980 (9)	0.0247 (4)	
H29A	−0.0349	0.5731	0.3915	0.030*	
C30	0.03784 (13)	0.7509 (2)	0.45716 (9)	0.0246 (4)	
C31	0.11572 (13)	0.7859 (2)	0.51189 (9)	0.0239 (4)	
H31A	0.1265	0.8982	0.5292	0.029*	
C32	0.17830 (13)	0.6522 (2)	0.54108 (8)	0.0210 (4)	
H32A	0.2332	0.6735	0.5788	0.025*	

### Atomic displacement parameters (Å^2^)

**Table:**

	*U*^11^	*U*^22^	*U*^33^	*U*^12^	*U*^13^	*U*^23^
F1	0.0311 (7)	0.0361 (6)	0.0309 (7)	0.0124 (5)	0.0036 (5)	0.0117 (5)
O1	0.0139 (6)	0.0242 (6)	0.0226 (7)	0.0003 (5)	0.0068 (5)	0.0041 (5)
O2	0.0162 (6)	0.0261 (6)	0.0231 (7)	0.0044 (5)	0.0097 (5)	0.0004 (5)
O3	0.0148 (6)	0.0168 (6)	0.0249 (7)	0.0031 (5)	0.0082 (5)	0.0019 (5)
N1	0.0177 (7)	0.0118 (6)	0.0132 (7)	−0.0006 (5)	0.0060 (6)	0.0002 (5)
N2	0.0198 (8)	0.0215 (7)	0.0194 (8)	−0.0020 (6)	0.0083 (6)	−0.0038 (6)
C1	0.0131 (8)	0.0136 (7)	0.0130 (8)	0.0008 (6)	0.0049 (6)	0.0015 (6)
C2	0.0146 (8)	0.0132 (7)	0.0151 (8)	0.0009 (6)	0.0047 (6)	−0.0013 (6)
C3	0.0164 (8)	0.0138 (7)	0.0118 (7)	0.0004 (6)	0.0042 (6)	−0.0008 (6)
C4	0.0214 (9)	0.0162 (8)	0.0138 (8)	0.0022 (7)	0.0039 (7)	−0.0014 (6)
C5	0.0305 (10)	0.0135 (8)	0.0141 (8)	0.0027 (7)	0.0044 (7)	0.0019 (6)
C6	0.0372 (11)	0.0181 (8)	0.0165 (9)	−0.0046 (8)	0.0128 (8)	0.0015 (7)
C7	0.0230 (10)	0.0205 (8)	0.0199 (9)	−0.0025 (7)	0.0115 (7)	−0.0004 (7)
C8	0.0176 (8)	0.0154 (7)	0.0126 (8)	−0.0015 (6)	0.0056 (6)	−0.0019 (6)
C9	0.0136 (8)	0.0177 (8)	0.0158 (8)	−0.0008 (6)	0.0051 (7)	−0.0006 (6)
C10	0.0109 (8)	0.0131 (7)	0.0149 (8)	0.0008 (6)	0.0050 (6)	0.0010 (6)
C11	0.0152 (8)	0.0149 (7)	0.0118 (8)	0.0006 (6)	0.0051 (6)	0.0011 (6)
C12	0.0142 (8)	0.0163 (8)	0.0159 (8)	−0.0016 (6)	0.0059 (7)	−0.0009 (6)
C13	0.0186 (9)	0.0161 (8)	0.0197 (9)	−0.0007 (7)	0.0085 (7)	−0.0006 (7)
C14	0.0238 (10)	0.0186 (8)	0.0266 (10)	−0.0068 (7)	0.0118 (8)	−0.0024 (7)
C15	0.0152 (9)	0.0294 (9)	0.0233 (9)	−0.0054 (7)	0.0078 (7)	−0.0016 (7)
C16	0.0136 (8)	0.0244 (9)	0.0187 (8)	0.0008 (7)	0.0055 (7)	−0.0005 (7)
C17	0.0143 (8)	0.0179 (8)	0.0132 (8)	0.0001 (6)	0.0059 (6)	−0.0003 (6)
C18	0.0135 (8)	0.0157 (8)	0.0163 (8)	0.0019 (6)	0.0047 (6)	0.0024 (6)
C19	0.0122 (8)	0.0150 (7)	0.0147 (8)	−0.0001 (6)	0.0060 (6)	0.0006 (6)
C20	0.0177 (9)	0.0149 (7)	0.0138 (8)	−0.0027 (6)	0.0057 (7)	0.0010 (6)
C21	0.0203 (9)	0.0185 (8)	0.0150 (8)	−0.0016 (7)	0.0043 (7)	−0.0013 (6)
C22	0.0137 (8)	0.0179 (8)	0.0150 (8)	−0.0009 (6)	0.0072 (7)	0.0003 (6)
C23	0.0153 (9)	0.0182 (8)	0.0193 (9)	−0.0005 (7)	0.0082 (7)	−0.0012 (7)
C24	0.0171 (9)	0.0231 (8)	0.0152 (8)	−0.0044 (7)	0.0059 (7)	−0.0027 (7)
C25	0.0135 (8)	0.0183 (8)	0.0162 (8)	−0.0038 (6)	0.0066 (7)	0.0002 (6)
C26	0.0165 (8)	0.0157 (8)	0.0160 (8)	−0.0001 (6)	0.0074 (7)	−0.0013 (6)
C27	0.0150 (8)	0.0220 (8)	0.0149 (8)	−0.0016 (7)	0.0070 (7)	0.0020 (7)
C28	0.0191 (9)	0.0278 (9)	0.0149 (8)	−0.0007 (7)	0.0063 (7)	0.0001 (7)
C29	0.0187 (9)	0.0371 (10)	0.0157 (9)	0.0010 (8)	0.0039 (7)	0.0028 (8)
C30	0.0212 (10)	0.0280 (9)	0.0229 (10)	0.0075 (8)	0.0065 (8)	0.0117 (8)
C31	0.0223 (10)	0.0207 (9)	0.0270 (10)	0.0004 (7)	0.0075 (8)	0.0053 (7)
C32	0.0188 (9)	0.0225 (9)	0.0187 (9)	−0.0021 (7)	0.0038 (7)	0.0037 (7)

### Geometric parameters (Å, °)

**Table:**

F1—C30	1.3702 (19)	C15—C16	1.390 (2)
O1—C2	1.2200 (19)	C15—H15A	0.9500
O2—C9	1.2170 (19)	C16—C17	1.397 (2)
O3—C11	1.2196 (19)	C16—H16A	0.9500
N1—C1	1.4537 (19)	C17—C18	1.506 (2)
N1—C21	1.458 (2)	C18—H18A	0.9900
N1—C20	1.476 (2)	C18—H18B	0.9900
N2—C24	1.341 (2)	C19—C22	1.515 (2)
N2—C23	1.344 (2)	C19—C20	1.549 (2)
C1—C9	1.541 (2)	C19—H19A	1.0000
C1—C2	1.554 (2)	C20—H20A	0.9900
C1—C10	1.574 (2)	C20—H20B	0.9900
C2—C3	1.475 (2)	C21—H21A	0.9800
C3—C4	1.393 (2)	C21—H21B	0.9800
C3—C8	1.394 (2)	C21—H21C	0.9800
C4—C5	1.388 (2)	C22—C26	1.391 (2)
C4—H4A	0.9500	C22—C23	1.395 (2)
C5—C6	1.401 (3)	C23—H23A	0.9500
C5—H5A	0.9500	C24—C25	1.399 (2)
C6—C7	1.393 (2)	C24—H24A	0.9500
C6—H6A	0.9500	C25—C26	1.396 (2)
C7—C8	1.392 (2)	C25—C27	1.485 (2)
C7—H7A	0.9500	C26—H26A	0.9500
C8—C9	1.481 (2)	C27—C28	1.400 (2)
C10—C11	1.545 (2)	C27—C32	1.401 (2)
C10—C18	1.548 (2)	C28—C29	1.388 (2)
C10—C19	1.562 (2)	C28—H28A	0.9500
C11—C12	1.471 (2)	C29—C30	1.371 (3)
C12—C17	1.391 (2)	C29—H29A	0.9500
C12—C13	1.403 (2)	C30—C31	1.372 (3)
C13—C14	1.384 (2)	C31—C32	1.388 (2)
C13—H13A	0.9500	C31—H31A	0.9500
C14—C15	1.399 (2)	C32—H32A	0.9500
C14—H14A	0.9500		
			
C1—N1—C21	115.76 (13)	C12—C17—C18	111.79 (14)
C1—N1—C20	106.41 (12)	C16—C17—C18	128.35 (15)
C21—N1—C20	115.26 (13)	C17—C18—C10	104.82 (12)
C24—N2—C23	117.35 (15)	C17—C18—H18A	110.8
N1—C1—C9	115.33 (13)	C10—C18—H18A	110.8
N1—C1—C2	115.67 (13)	C17—C18—H18B	110.8
C9—C1—C2	101.66 (12)	C10—C18—H18B	110.8
N1—C1—C10	99.85 (12)	H18A—C18—H18B	108.9
C9—C1—C10	112.61 (13)	C22—C19—C20	116.22 (13)
C2—C1—C10	112.26 (12)	C22—C19—C10	113.93 (13)
O1—C2—C3	126.58 (15)	C20—C19—C10	104.16 (13)
O1—C2—C1	125.69 (15)	C22—C19—H19A	107.4
C3—C2—C1	107.60 (13)	C20—C19—H19A	107.4
C4—C3—C8	121.34 (15)	C10—C19—H19A	107.4
C4—C3—C2	128.58 (16)	N1—C20—C19	105.02 (12)
C8—C3—C2	110.06 (14)	N1—C20—H20A	110.7
C5—C4—C3	117.37 (17)	C19—C20—H20A	110.7
C5—C4—H4A	121.3	N1—C20—H20B	110.7
C3—C4—H4A	121.3	C19—C20—H20B	110.7
C4—C5—C6	121.71 (16)	H20A—C20—H20B	108.8
C4—C5—H5A	119.1	N1—C21—H21A	109.5
C6—C5—H5A	119.1	N1—C21—H21B	109.5
C7—C6—C5	120.53 (16)	H21A—C21—H21B	109.5
C7—C6—H6A	119.7	N1—C21—H21C	109.5
C5—C6—H6A	119.7	H21A—C21—H21C	109.5
C8—C7—C6	117.91 (16)	H21B—C21—H21C	109.5
C8—C7—H7A	121.0	C26—C22—C23	116.61 (15)
C6—C7—H7A	121.0	C26—C22—C19	123.18 (14)
C7—C8—C3	121.11 (15)	C23—C22—C19	120.18 (14)
C7—C8—C9	129.14 (16)	N2—C23—C22	124.41 (16)
C3—C8—C9	109.66 (14)	N2—C23—H23A	117.8
O2—C9—C8	126.60 (15)	C22—C23—H23A	117.8
O2—C9—C1	125.80 (15)	N2—C24—C25	123.58 (16)
C8—C9—C1	107.55 (13)	N2—C24—H24A	118.2
C11—C10—C18	104.44 (12)	C25—C24—H24A	118.2
C11—C10—C19	114.49 (13)	C26—C25—C24	117.20 (15)
C18—C10—C19	116.40 (13)	C26—C25—C27	120.58 (15)
C11—C10—C1	111.14 (13)	C24—C25—C27	122.22 (15)
C18—C10—C1	111.13 (13)	C22—C26—C25	120.82 (15)
C19—C10—C1	99.42 (12)	C22—C26—H26A	119.6
O3—C11—C12	127.60 (15)	C25—C26—H26A	119.6
O3—C11—C10	124.71 (15)	C28—C27—C32	118.59 (16)
C12—C11—C10	107.67 (13)	C28—C27—C25	121.06 (15)
C17—C12—C13	121.85 (15)	C32—C27—C25	120.32 (15)
C17—C12—C11	109.63 (14)	C29—C28—C27	120.67 (17)
C13—C12—C11	128.51 (15)	C29—C28—H28A	119.7
C14—C13—C12	117.71 (16)	C27—C28—H28A	119.7
C14—C13—H13A	121.1	C30—C29—C28	118.33 (17)
C12—C13—H13A	121.1	C30—C29—H29A	120.8
C13—C14—C15	120.88 (16)	C28—C29—H29A	120.8
C13—C14—H14A	119.6	F1—C30—C29	118.38 (16)
C15—C14—H14A	119.6	F1—C30—C31	118.15 (17)
C16—C15—C14	121.10 (16)	C29—C30—C31	123.47 (17)
C16—C15—H15A	119.5	C30—C31—C32	117.89 (17)
C14—C15—H15A	119.5	C30—C31—H31A	121.1
C15—C16—C17	118.59 (16)	C32—C31—H31A	121.1
C15—C16—H16A	120.7	C31—C32—C27	121.03 (17)
C17—C16—H16A	120.7	C31—C32—H32A	119.5
C12—C17—C16	119.84 (15)	C27—C32—H32A	119.5
			
C21—N1—C1—C9	62.03 (19)	C10—C11—C12—C13	−171.27 (16)
C20—N1—C1—C9	−168.47 (13)	C17—C12—C13—C14	0.6 (3)
C21—N1—C1—C2	−56.37 (19)	C11—C12—C13—C14	179.77 (16)
C20—N1—C1—C2	73.13 (16)	C12—C13—C14—C15	0.8 (3)
C21—N1—C1—C10	−177.04 (13)	C13—C14—C15—C16	−0.8 (3)
C20—N1—C1—C10	−47.54 (15)	C14—C15—C16—C17	−0.4 (3)
N1—C1—C2—O1	−34.0 (2)	C13—C12—C17—C16	−1.9 (3)
C9—C1—C2—O1	−159.71 (16)	C11—C12—C17—C16	178.80 (15)
C10—C1—C2—O1	79.72 (19)	C13—C12—C17—C18	179.42 (15)
N1—C1—C2—C3	142.07 (13)	C11—C12—C17—C18	0.13 (19)
C9—C1—C2—C3	16.34 (16)	C15—C16—C17—C12	1.8 (2)
C10—C1—C2—C3	−104.23 (15)	C15—C16—C17—C18	−179.80 (16)
O1—C2—C3—C4	−11.5 (3)	C12—C17—C18—C10	−8.03 (18)
C1—C2—C3—C4	172.51 (15)	C16—C17—C18—C10	173.44 (16)
O1—C2—C3—C8	167.33 (16)	C11—C10—C18—C17	12.09 (16)
C1—C2—C3—C8	−8.67 (18)	C19—C10—C18—C17	139.37 (14)
C8—C3—C4—C5	1.0 (2)	C1—C10—C18—C17	−107.82 (14)
C2—C3—C4—C5	179.71 (16)	C11—C10—C19—C22	82.84 (17)
C3—C4—C5—C6	0.3 (2)	C18—C10—C19—C22	−39.30 (19)
C4—C5—C6—C7	−1.2 (3)	C1—C10—C19—C22	−158.66 (13)
C5—C6—C7—C8	0.8 (3)	C11—C10—C19—C20	−149.54 (13)
C6—C7—C8—C3	0.5 (2)	C18—C10—C19—C20	88.32 (16)
C6—C7—C8—C9	−175.63 (16)	C1—C10—C19—C20	−31.03 (14)
C4—C3—C8—C7	−1.4 (2)	C1—N1—C20—C19	27.63 (16)
C2—C3—C8—C7	179.65 (15)	C21—N1—C20—C19	157.41 (13)
C4—C3—C8—C9	175.39 (14)	C22—C19—C20—N1	130.35 (14)
C2—C3—C8—C9	−3.53 (18)	C10—C19—C20—N1	4.16 (16)
C7—C8—C9—O2	13.4 (3)	C20—C19—C22—C26	−31.0 (2)
C3—C8—C9—O2	−163.05 (16)	C10—C19—C22—C26	90.10 (18)
C7—C8—C9—C1	−169.08 (16)	C20—C19—C22—C23	150.83 (15)
C3—C8—C9—C1	14.43 (18)	C10—C19—C22—C23	−88.05 (18)
N1—C1—C9—O2	33.2 (2)	C24—N2—C23—C22	−1.3 (2)
C2—C1—C9—O2	159.15 (16)	C26—C22—C23—N2	1.8 (2)
C10—C1—C9—O2	−80.52 (19)	C19—C22—C23—N2	−179.93 (15)
N1—C1—C9—C8	−144.31 (14)	C23—N2—C24—C25	−0.5 (2)
C2—C1—C9—C8	−18.35 (16)	N2—C24—C25—C26	1.6 (2)
C10—C1—C9—C8	101.97 (15)	N2—C24—C25—C27	−177.84 (15)
N1—C1—C10—C11	168.49 (13)	C23—C22—C26—C25	−0.5 (2)
C9—C1—C10—C11	−68.64 (16)	C19—C22—C26—C25	−178.74 (15)
C2—C1—C10—C11	45.39 (17)	C24—C25—C26—C22	−1.0 (2)
N1—C1—C10—C18	−75.66 (15)	C27—C25—C26—C22	178.41 (15)
C9—C1—C10—C18	47.21 (17)	C26—C25—C27—C28	−145.25 (17)
C2—C1—C10—C18	161.23 (13)	C24—C25—C27—C28	34.2 (2)
N1—C1—C10—C19	47.52 (14)	C26—C25—C27—C32	32.6 (2)
C9—C1—C10—C19	170.39 (12)	C24—C25—C27—C32	−147.97 (17)
C2—C1—C10—C19	−75.59 (14)	C32—C27—C28—C29	−1.2 (3)
C18—C10—C11—O3	169.18 (15)	C25—C27—C28—C29	176.67 (16)
C19—C10—C11—O3	40.7 (2)	C27—C28—C29—C30	0.0 (3)
C1—C10—C11—O3	−70.9 (2)	C28—C29—C30—F1	−178.37 (16)
C18—C10—C11—C12	−12.43 (17)	C28—C29—C30—C31	0.9 (3)
C19—C10—C11—C12	−140.87 (14)	F1—C30—C31—C32	178.73 (16)
C1—C10—C11—C12	107.48 (15)	C29—C30—C31—C32	−0.6 (3)
O3—C11—C12—C17	−173.71 (16)	C30—C31—C32—C27	−0.7 (3)
C10—C11—C12—C17	7.96 (18)	C28—C27—C32—C31	1.6 (3)
O3—C11—C12—C13	7.1 (3)	C25—C27—C32—C31	−176.31 (16)

### Hydrogen-bond geometry (Å, °)

**Table:**

Cg1 is the centroid of the N2/C22–C26 pyridyl ring.

**Table:**

*D*—H···*A*	*D*—H	H···*A*	*D*···*A*	*D*—H···*A*
C6—H6A···N2^i^	0.95	2.56	3.476 (2)	163
C13—H13A···N1^ii^	0.95	2.45	3.387 (2)	168
C15—H15A···O2^iii^	0.95	2.44	3.381 (3)	169
C18—H18A···O2	0.99	2.40	3.077 (2)	125
C20—H20A···O3^iv^	0.99	2.55	3.318 (2)	135
C4—H4A···Cg1^v^	0.95	2.65	3.552 (2)	159

Symmetry codes: (i) *x*, −*y*−1/2, *z*+1/2; (ii) *x*, *y*−1, *z*; (iii) −*x*, *y*−1/2, −*z*+3/2; (iv) −*x*+1, *y*+1/2, −*z*+3/2; (v) −*x*+1, *y*−1/2, −*z*+3/2.
